# Test-retest repeatability of ADC in prostate using the multi *b*-Value VERDICT acquisition

**DOI:** 10.1016/j.ejrad.2023.110782

**Published:** 2023-03-16

**Authors:** Harriet J. Rogers, Saurabh Singh, Anna Barnes, Nancy A. Obuchowski, Daniel J. Margolis, Dariya I. Malyarenko, Thomas L. Chenevert, Amita Shukla-Dave, Michael A. Boss, Shonit Punwani

**Affiliations:** a Centre for Medical Imaging, Division of Medicine, University College London, London, UK; b Department of Quantitative Health Sciences, Cleveland Clinic Foundation, Cleveland, OH, USA; c Radiology, Weill Cornell Medicine, New York, NY, USA; d Department of Radiology, University of Michigan, Ann Arbor, MI, USA; e Departments of Medical Physics and Radiology, Memorial Sloan Kettering Cancer Center, New York, NY, USA; f Center for Research and Innovation, American College of Radiology, Philadelphia, PA, USA

**Keywords:** Magnetic Resonance Imaging, Prostate Cancer, Diffusion Weighted Imaging, Apparent Diffusion Coefficient, Repeatability

## Abstract

**Purpose:**

VERDICT (Vascular, Extracellular, Restricted Diffusion for Cytometry in Tumours) MRI is a multi *b*-value, variable diffusion time DWI sequence that allows generation of ADC maps from different *b*-value and diffusion time combinations. The aim was to assess precision of prostate ADC measurements from varying *b*-value combinations using VERDICT and determine which protocol provides the most repeatable ADC.

**Materials and Methods:**

Forty-one men (median age: 67.7 years) from a prior prospective VERDICT study (April 2016–October 2017) were analysed retrospectively. Men who were suspected of prostate cancer and scanned twice using VERDICT were included. ADC maps were formed using 5*b*-value combinations and the within-subject standard deviations (wSD) were calculated per ADC map. Three anatomical locations were analysed per subject: normal TZ (transition zone), normal PZ (peripheral zone), and index lesions. Repeated measures ANOVAs showed which *b*-value range had the lowest wSD, Spearman correlation and generalized linear model regression analysis determined whether wSD was related to ADC magnitude and ROI size.

**Results:**

The mean lesion ADC for *b*_0_ *b*_1500_ had the lowest wSD in most zones (0.18–0.58x10^−4^ mm^2^/s). The wSD was unaffected by ADC magnitude (Lesion: *p* = 0.064, TZ: *p* = 0.368, PZ: *p* = 0.072) and lesion Likert score (*p* = 0.95). wSD showed a decrease with ROI size pooled over zones (*p* = 0.019, adjusted regression coefficient = −1.6x10^−3^, larger ROIs for TZ versus PZ versus lesions). ADC maps formed with a maximum *b*-value of 500 s/mm^2^ had the largest wSDs (1.90–10.24x10^−4^ mm^2^/s).

**Conclusion:**

ADC maps generated from *b*_0_ *b*_1500_ have better repeatability in normal TZ, normal PZ, and index lesions.

## 1. Introduction

Multiparametric MRI (mpMRI) is an established imaging technique for investigating suspected prostate cancer [1]. Diffusion Weighted Imaging (DWI) and the derived Apparent Diffusion Coefficient (ADC) maps are a central part of the mpMRI protocol. PI-RADS V2.1 (Prostate Imaging-Reporting and Data System) acknowledge that ADC is often calculated from ≥ 2*b*-values and that ADC is affected by *b*-value choice [2]. If only 2*b*-values are acquired, the recommendation is to use 100 s/mm^2^ (preferably 50–100 s/mm^2^) for the lowest and 800–1000 s/mm^2^ for b_*max*_. This range is achievable by most clinical scanners and should avoid the diffusion kurtosis effect, seen at higher *b*-values. Due to a lack of ADC repeatability and reproducibility data, current PI-RADS uses ADC only as a qualitative component of prostate cancer risk stratification. Recently implemented guidelines by European Association of Urology [3] (EAU, 2019) advocate the use of quantitative ADC thresholds for detection of low-grade cancer to allow ~ 30% reduction in unnecessary biopsies [4].

For quantitative prostate ADC use, QIBA (Quantitative Imaging Biomarkers Alliance) suggests that 2*b*-values are included: lower *b*-values: 50–100 s/mm^2^ and *b_max_* = 500–1500 s/mm^2^ [5]. QIBA stresses the importance of assessing the precision (repeatability) of ADC to define confidence intervals (CIs) for quantitative ADC thresholds, which can be determined through calculations of the wSD (within-subject standard deviation) of test–retest scans [6]. For longitudinal studies, e.g. assessing therapy response, wSD can be used to determine the significant changes in ADC [5,6]. To establish CI for cross-sectional, diagnostic thresholds [5,6] estimate of bias (accuracy) would be required in addition to repeatability. The range and combination of *b*-values have an impact on prostate ADC, with significant differences observed from ADC from 2*b* to values compared to multiple *b*-values [7,8]. Furthermore, *b_max_* may impact clinical utility; one study showed greater accuracy at predicting prostate cancer with ADC from *b*_*max*_ = *b*_1000_ compared to *b*_*max*_ = *b*_2000_, whereas the opposite has also been demonstrated [9]. Since ADC values are varying among the prostate zones and lesions (e.g., lower for TZ and cancer), different *b*-value combinations are expected to have varying impact on ADC contrast and repeatability in different prostate locations.

To date, a handful of rigorous prostate ADC repeatability studies, with limited subject numbers (<12) [10–12], have shown low prostate ADC repeatability (~50%) compared to ADC of other organs [5]. QIBA recommends > 35 subjects for test–retest studies to achieve nominal confidence intervals for repeatability estimate [13]. The larger multicentre ADC repeatability study of 29 subjects in the imaging arm of ACRIN 6701 trial [14], has recently reported encouraging observations of better than 10% ADC (*b* = 0,800 s/mm^2^) repeatability for the whole prostate scanned on the same day or up to two weeks apart, but included evaluation for only 10 lesions with much lower reported ADC repeatability (up to 40%). Due to small subject numbers and challenges of DWI prostate acquisition and analysis, previous studies were not statistically powered to assess the dependence of ADC repeatability on acquisition protocol (*b*-values and diffusion times) and analysis (lesion size and location). Such dependencies are of interest for radiologists to improve prostate ADC precision and enable the inclusion of quantitative ADC information in future PI-RADS guidelines.

VERDICT (Vascular, Extracellular, Restricted Diffusion for Cytometry in Tumours) is an advanced diffusion imaging technique that utilises six *b*-values (0 90 500 1500 2000 3000 s/mm^2^) with varying diffusion times, and a mathematical model to derive quantitative intra- and extra-cellular tissue fractions [15]. VERDICT derived parameters have demonstrated clinical promise to distinguish between high and low grade prostate cancer [16], and to avoid unnecessary biopsies [17]. The acquired diffusion data also allows generation of ADC maps using different permutations of *b*-values and diffusion times. Given the potential clinical utility, and that the sequence consists of multiple *b*-values, it would be beneficial to establish if a repeatable ADC could also be derived from the VERDICT scan, as opposed to acquiring an additional DWI sequence separately.

Detection of clinically significant changes in ADC requires determination of the baseline variability in ADC measurements [6]. This study sought to establish which *b*-value combination provides the most repeatable prostate ADC, using the VERDICT acquisition for test–retest assessment of > 35 subjects.

## 2. Materials and Methods

The present study retrospectively analysed data from a repeatability arm (*n* = 41) of a prospective cohort study (*n* = 70) called INNOVATE (ClinicalTrials.gov identifier NCT02689271) which focused on the diagnostic utility of VERDICT parameters, rather than ADC repeatability due to varying diffusion times for different *b*-value combinations [16]. Ethical approval was obtained from the London–Surrey Borders Research Ethics Committee and written informed consent was attained from study participants. The full study protocol is available at [18]. Prostate Cancer UK funded the trial.

### 2.1. Study participants

Men were eligible if they had clinical suspicion of prostate cancer due to either a raised PSA or a suspicious digital rectal examination, or were undergoing active surveillance. Participants were excluded if they had undergone prostate cancer treatment, were receiving ongoing hormonal prostate cancer treatment, or had received a biopsy within 6 months before their MRI.

The repeatability cohort included 41 men who had two identical multi *b*-value VERDICT acquisitions with an interval ≤ 5 min (median age: 67.7 years; range: 50–82 years). Of these 41, 10 men were randomly selected to vacate the scanner after their first scan which necessitated repositioning for the second acquisition. The remaining 31 stayed in the scanner between acquisitions and were not repositioned. Patient demographics are listed in Table 1. Analysis was separated for the 2 repositioning groups. All men underwent single clinical mpMRI in addition to the repeated multi *b*-value VERDICT acquisition on a 3 T MRI scanner. The mpMRI was assessed by experienced uro-radiologists (>10 years’ experience in mpMRI).

Following mpMRI, 19 men with a PI-RADS score ≥ 3 had targeted transperineal biopsy of identified lesions. Histologic examinations from the biopsy cores were evaluated in the standard clinical fashion and assigned an overall Gleason Grade [19](A.F. and M.R, 13- and 15-years prostate pathology experience, respectively).

### 2.2. MR Imaging and image analysis

All imaging was performed on a 3 T Philips Achieva scanner. Hyoscine butylbromide (Buscopan, Boehringer Ingelheim, Ingelheim am Rhein, Germany; 0.2 mg/kg, up to 20 mg) was intravenously administered prior to imaging to reduce peristalsis.

Details of the multi *b*-value VERDICT sequence are in Table 2 (Acquisition Time: 12 min 25 s). Patients also underwent mpMRI in the same session [20].

ADC maps were generated from subsets of the multi *b*-value VERDICT data. Trace images were generated for each *b*-value. These were registered to *b*_0_ from *b*_3000_ from the first acquisition for each subject. ADC maps were generated using an in-house model designed in MATLAB (version 2020a, MathWorks Inc., Natick, MA, USA) on a voxel-by-voxel basis using Eq. (1), where *S(b)* is the signal at a given *b*-value, and *S_0_* is the signal with no diffusion weighting.


(1) $$S(b)=S_{0}e^{-\mathrm{b.ADC}}$$

Various *b*-value combinations were used to generate ADC maps to test ADC repeatability. In total, 5 versions of ADC maps were created for each subject for scan1 and scan2 (Table 3). These 5*b*-value combinations were selected from all available to ensure there was ≤ 15 ms difference between the scan TEs (to alleviate inconsistent T_2_ weighting for different *b*-values intrinsic to the VERDICT protocol, Table 2) and excluded b_*3000*_ which may not be clinically achievable. TE = 80 ms was used for all combinations which included *b*_0_, (the *b*_0_ image from the *b*_*max*_ = 3000 acquisition from the full VERDICT sequence).

Regions of Interest (ROIs) were created using Mango Software (Research Imaging Institute, UTHSCSA). ROIs were drawn in 3 locations per subject: around the index lesion identified on mpMRI, in normal TZ and normal PZ, by an experienced board-certified radiologist (S.S 4 years’ experience in mpMRI). As all trace images were registered to the *b*_0_ from *b*_3000_ from the first acquisition per subject, only one set of ROIs was needed per subject. These ROIs were then applied onto the registered ADC maps for scan2. Normal PZ and TZ were selected based on PI-RADS scores of 1 or 2. Analyses were confined within the ROIs; therefore, if ROI delineation was not possible within a certain location it was excluded for that subject. Furthermore, image quality was visually assessed and if an ROI showed poor quality or artefacts it was also removed from analysis.

### 2.3. Statistical analysis

Statistical analysis was conducted in SPSS (version 24 [IBM, Armonk, NY]) and SAS, and hypothesis tests were two-sided. The assumption of normality for wSD of the ADC was checked by performing a Wilks Shapiro test, and also by examining the mean, median, and mode of the distribution, as well as skewness and kurtosis. To assess repeatability, the within-subject standard deviation (wSD) [5] was calculated for each subject and each ADC map in all 3 locations (normal TZ, normal PZ, and index lesions). This was achieved by calculating the variance for each subject, given by the squared difference between both measurements divided by 2 and taking the mean of the variances across all subjects (providing the within-subject variance), then taking the square root of this value.

The wSD was calculated for all five ADC maps per region. The distributions of wSD, wSD^2^, and the log distributions were compared and the robustness of the wSD results was assessed with a model based on the logs. To test if repeatability was affected by the magnitude of the ADC value, for each *b*-value combination, GLMs (Generalized Linear Models) were fit where the dependent variable was the wSD and the independent variable was the magnitude of the ADC (using ADC mean from scan1 and scan2). To determine which *b*-value range provided the lowest wSD, repeated measure ANOVAs with Bonferroni adjustments for multiple comparisons were conducted for the separate locations. GLMs were performed for each region (TZ, PZ and index lesions). First, the interaction between *b*-value combination and whether or not the patient was repositioned was tested. If statistically significant (*p* < 0.05), these subjects were analysed separately, if not significant, the data was pooled and adjusted for the binary variable of whether or not the patient had been repositioned. A statistically significant difference between wSD values was then considered if *p* < 0.05.

Bland-Altman plots were used to identify trends in the differences and construct limits of agreement (LOA) between the two repeated scans. Additionally, Spearman correlation coefficients were used to assess the association between ADC mean and Lesion Likert score. The associations based on the correlation coefficients were interpreted as: 0.00–0.20 = negligible, 0.21–0.40 = weak, 0.41–0.60 = moderate, 0.61–0.8 = strong, and 0.81–1.00 = very strong. A statistically significant relationship was considered if *p* < 0.05.

To assess the effect of ROI size on repeatability, the data from normal TZ, normal PZ and index lesions were pooled. A GLM was fit where the dependent variable was the log of wSD and the independent variables were location (normal TZ, normal PZ, or index lesion) and voxel count within ROIs. We used GEEs (Generalized Estimating Equations) to account for the clustered nature of the data, treating patients as a random effect and with an exchangeable working correlation matrix structure, and assessed adjusted regression coefficient for ROI voxel count. A Wald test was used to determine if the regression coefficient for number of voxels was significantly different from zero. A statistically significant difference was considered if *p* < 0.05.

## 3. Results

**Fig. 1.**
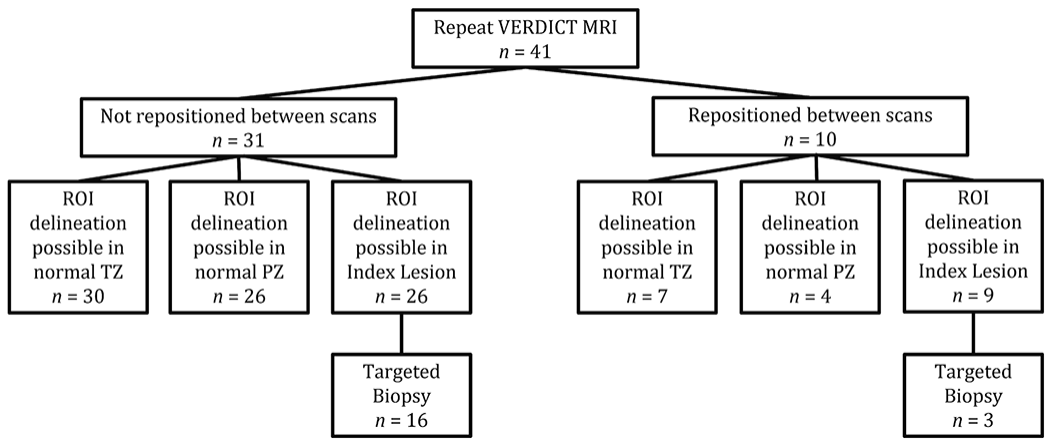
Flow diagram detailing included participants. VERDICT MRI = Advanced Diffusion MRI sequence (Vascular, Extracellular, Restricted Diffusion for Cytometry in Tumours). Normal TZ = Normal Transition Zone. Normal PZ = Normal Peripheral Zone.

A total of 41 men were included in the short-term repeatability study (31 who did not undergo repositioning between scan1 and scan2, and 10 who were randomly assigned to be repositioned). A flow chart detailing the patient numbers is shown in Fig. 1. In total, ADC repeatability was studied for 37 prostate regions in normal TZ, 30 in normal PZ and 35 index lesions (19 biopsied).

**Fig. 2.**
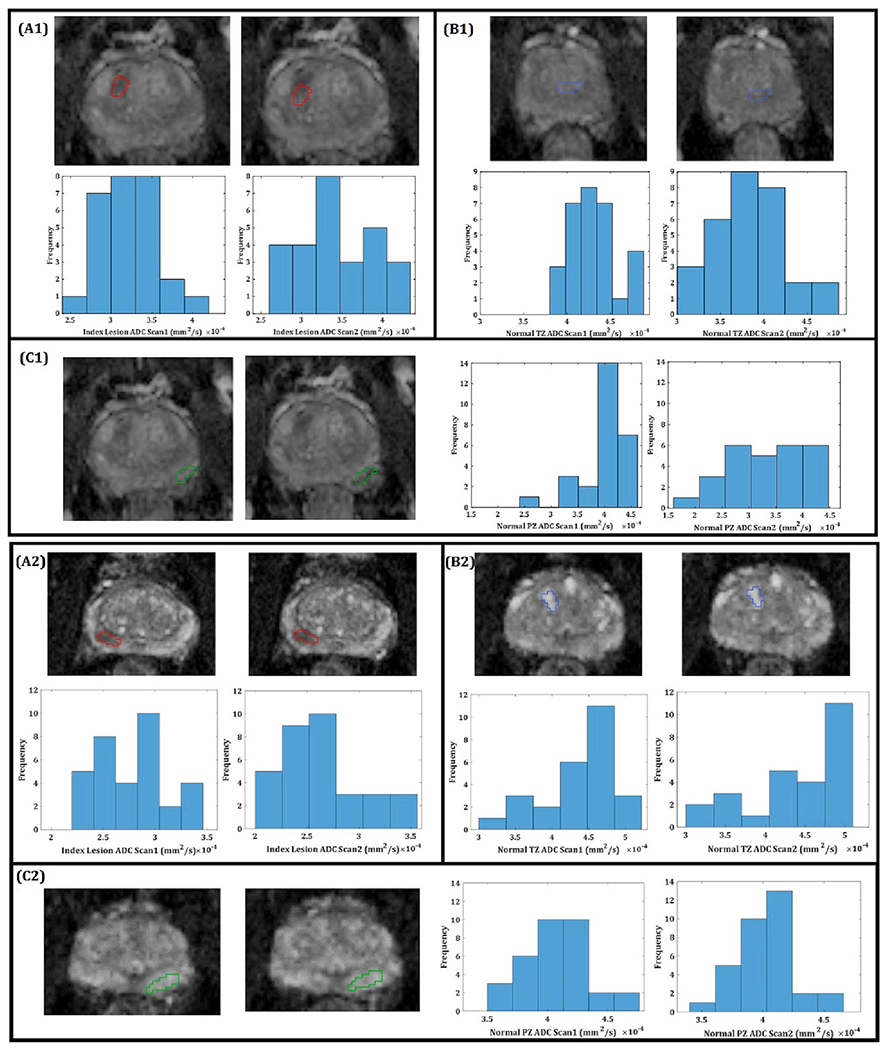
ADC (Apparent Diffusion Coefficient) maps from two representative patients who was scanned twice back-to-back with no repositioning. ADC maps generated from *b*_0_ *b*_1500_ s/mm^2^. The panels show the ROIs and corresponding histograms with the distribution of the ADC values for the first and second scans. Top panel index lesion Likert: 4, Gleason Score: 3 + 4. Bottom panel index lesion Likert: 5, Gleason Score: 4 + 3. (**A1**, **A2**) Outlined in red is the index lesion(**B1**, **B2**) Outlined in blue is an ROI for normal transition zone. (**C1**, **C2**) Outlined in green is an ROI for normal peripheral zone. (For interpretation of the references to color in this figure legend, the reader is referred to the web version of this article.)

Of the non-repositioned group, ROI delineation (e.g., Fig. 2) was possible in the normal TZ for 30 participants and in the normal PZ for 26 participants. Twenty-six men had index lesions which were scored using PI-RADS (PI-RADS Version 2.1). All index lesions scored PI-RADS ≥ 3 (14/26 (53.8%) = 3, 9/26 (34.6%) = 4, 3/26 (11.5%) = 5). Four index lesions were defined in the TZ (2/4 were in the non-repositioned cohort and 1 of these overlapped between the PZ and TZ). Sixteen of the 26 lesions underwent targeted biopsy: 6 had no significant cancer, and the remaining 10 had clinically significant prostate cancer with a Gleason Grade ≥ 3 + 4 (8/10 (80%) = 3 + 4, 1/10 (10%) = 4 + 3, 1/10 (10%) = 4 + 5). The PI-RADS scores for the biopsied lesions were as follows: 7/16 (43.8%) = PI-RADS 3, 6/16 (37.5%) = PI-RADS 4, and 3/16 (18.8%) = PI-RADS 5.

Of the 10 men who were repositioned between scans, ROI delineation was possible in the normal TZ for 7 participants and in the normal PZ for 4 participants. Nine men had index lesions which all had PI-RADS ≥ 3 (3/9 (33.3%) = 3, 4/9 (44.4%) = 4, 2/9 (22.2%) = 5). Three of the 9 lesions underwent targeted biopsy all with clinically significant prostate cancer with Gleason Grade ≥ 3 + 4 (1/3 (33.3%) = 3 + 4, 1/3 (33.3%) = 4 + 3, 1/3 (33.3%) = 4 + 5). The PI-RADS scores for the biopsied lesions were as follows: 2/3 (66.7%) = PI-RADS 4, 1/3 (33.3%) = PI-RADS 5.

Fig. 2 shows examples of scan1 and scan2 ADC maps calculated from *b*_0_ *b*_1500_ with a delineated index lesion and normal TZ and PZ regions, and the corresponding ADC histograms illustrating variability between test and retest measurements. Mean ADC and wSD values across all subjects for each of the 5*b*-value combinations are summarised in Table 4.

For index lesions, ADC showed a moderate negative statistically significant correlation with Likert score when generated from *b*_0_ *b*_1500_ (*r* = −0.52, *p* < 0.002) and *b*_0_ *b_500_ b*_2000_ (*r* = −0.56, *p* < 0.001), while ADC from *b*_0_ *b*_2000_ was less correlated with the Likert score (*r* = −0.34, *p* = 0.049). No statistically significant correlations were observed between Likert sore and ADC from *b*_0_ *b*_500_ (*r* = 0.054, *p* = 0.756) or *b*_90_ *b*_500_ (*r* = −0.05, *p* = 0.77). The mean ADC values for index lesions were lower than those for normal PZ and similar to normal TZ values.

In a model assessing the effect of ADC magnitude on wSD, no statistically significant relationship was found (*p* > 0.05), thus, estimates of wSD were constant over the range of ADC values, and so wSD was deemed a more appropriate repeatability metric than wCV.

**Fig. 3.**
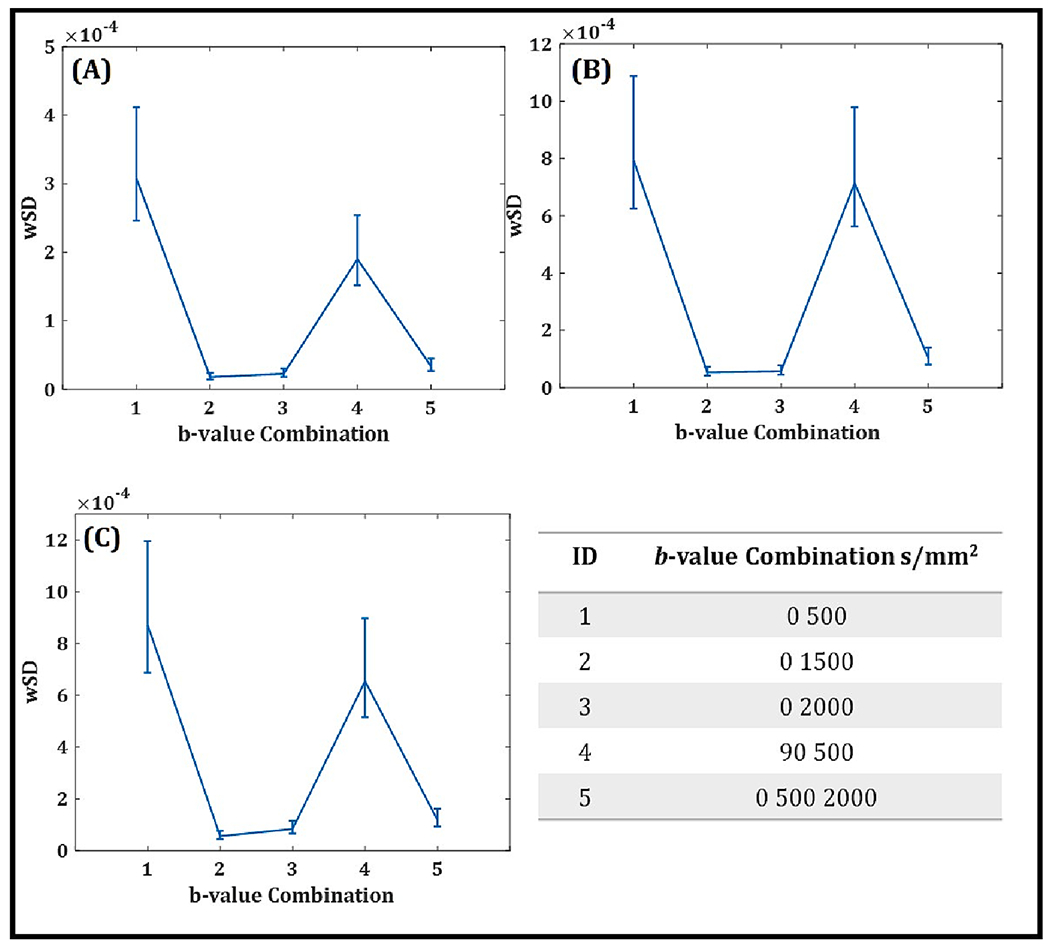
Wsd (within standard deviation) for each *b*-value combination ADC (Apparent Diffusion Coefficient) map with error bars indicating the confidence intervals for the non-repositioned cohort **(A)** Normal Transition Zone **(B)** Normal Peripheral Zone **(C)** Index Lesion.

Fig. 3 displays the wSD values and associated confidence intervals for the 5 different *b*-value combinations of the non-repositioned cohort. For index lesions and normal TZ, there were no statistically significant interactions between *b*-value combination and whether or not patients had undergone repositioning (index lesions: *p* = 0.222, TZ: *p* = 0.061), therefore the data was pooled. For normal PZ, this interaction was statistically significant (*p* = 0.010) therefore, the 26 patients who were not repositioned and the 4 who were repositioned, were analysed separately.

Across all regions, ADC with *b*_*max*_ = 500 s/mm^2^ had larger wSD values compared to all other ADC maps. In index lesions, normal TZ, and non-repositioned PZ, wSD was statistically significantly lower for *b*_0_ *b*_1500_, *b*_0_ *b*_2000_, and *b*_0_ *b*_500_ *b*_2000_, than for *b*_90_ *b*_500_ and *b*_0_ *b*_500_ (*p* = 0.001). For repositioned PZ, wSD was also statistically significantly lower for the *b*_0_ *b*_1500_ (*p* = 0.001) and *b*_0_ *b*_500_ *b*_2000_ (*p* = 0.016) compared to *b*_0_ *b*_500_. This was also the case for *b*_0_ *b*_1500_ (*p* = 0.011), *b*_0_ *b*_2000_ (*p* = 0.001) and *b*_0_ *b*_500_ *b*_2000_ (*p* = 0.032) compared to *b*_90_ *b*_500_. In all regions wSD was not statistically significantly different between *b*_90_ *b*_500_ and *b*_0_ *b*_500_ (*p* > 0.05).

The wSD of *b*_0_ *b*_1500_ was statistically significantly lower than *b*_0_ *b*_500_ *b*_2000_ in index lesions (*p* = 0.001) and the non-repositioned PZ (*p* = 0.001), however this difference did not reach statistical significance in the TZ (*p* = 0.118) or repositioned PZ (*p* = 0.813). As shown in Table 4, b0 b1500 provided the lowest wSD in all regions in the non-repositioned cohort and for index lesions within the repositioned cohort, while *b*_0_ *b*_2000_ provided the lowest wSD for normal PZ and TZ in the repositioned cohort. However, the difference between these wSDs never reached statistical significance (*p* > 0.05). Given that *b*_0_ *b*_1500_ provided the lowest wSD in the majority of regions, this *b*-value combination was used for further analysis.

**Fig. 4.**
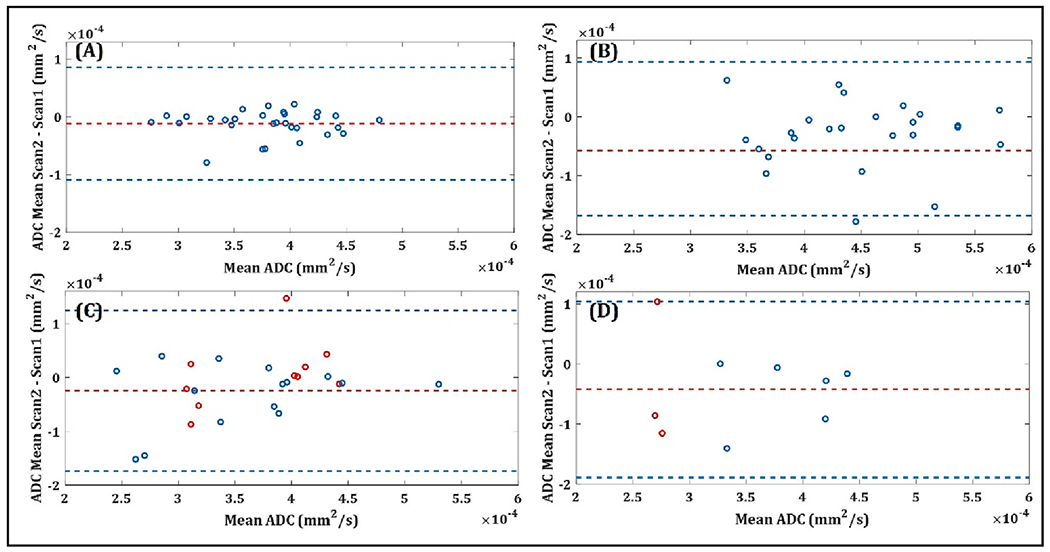
Bland-Altman analyses showing the level of agreement of mean ADC (Apparent Diffusion Coefficient) between scan 1 and scan 2. ADC generated from *b*_0_ *b*_1500_ s/mm^2^. Blue dotted lines indicate the limits of agreement (1.96*standard deviation). The Confidence Intervals (CI) for each of the analysed regions are provided: **(A)** Normal Transition Zone for non-repositioned cohort, (CI: [0.14, 0.24] x10^−4^ mm^2^/s) **(B)** Normal Peripheral Zone for non-repositioned cohort, (CI: [0.42, 0.73] x10^−4^ mm^2^/s) **(C)** Index Lesion for non-repositioned cohort (CI: [0.44, 0.76] x10^−4^ mm^2^/s) in red are the index lesions which were significant on biopsy (CI: [0.29, 0.74] x10^−4^ mm^2^/s) **(D)** Index Lesions for repositioned cohort (CI: [0.40, 1.06] x10^−4^ mm^2^/s), in red are the index lesions which were significant on biopsy (CI: [0.41, 2.70] x10^−4^ mm^2^/s). (For interpretation of the references to color in this figure legend, the reader is referred to the web version of this article.)

Bland-Altman analysis of ADC from *b*_0_ *b*_1500_ (Fig. 4) revealed narrow limits of agreement and a consistently small mean difference (bias) of<0.5 x10^−4^mm^2^/s between ADC values when comparing first and second scans in all regions. Mostly negative bias observed for PZ and index lesions suggested the tendency for lower ADC on the retest scan. (The Bland-Altman plots for other *b*-value combinations are provided for illustration in Supplementary Figures S1–S4.) Apparent test–retest variability (shown via the Bland-Altman confidence intervals) increased from normal TZ (0.98 x10^−4^mm^2^/s) versus normal PZ (1.31 x10^−4^ mm^2^/s) versus lesions (1.49 x10^−4^mm^2^/s). The index lesions from the repositioned group showed a very similar pattern to the index lesions from the non-repositioned group with a mean difference (bias) of −0.42 x10^−4^mm^2^/s and an apparent test–retest variability of 1.46 x10^−4^mm^2^/s (Fig. 4d). Additionally, lesion wSD for *b*_0_ *b*_1500_ did not show a statistically significant correlation to Likert score (*p* = 0.95).

Pooling the data for repositioned (n = 9) and non-repositioned (n = 26) index lesions (Fig. 4c,d) provided an average wSD of 0.39x10^−4^mm^2^/s for *b*_0_ *b*_1500_. This was closely comparable to the wSD (0.39x10^−4^mm^2^/s) of the biopsied cohort with confirmed significant cancer (Gleason Grade ≥ 3 + 4), which included 3 repositioned and 10 non-repositioned cases (color-coded in Fig. 4c,d). For 7 of the biopsy-confirmed significant cancers, average test–retest ADC was below minimum observed for normal PZ (<0.32x10^−3^mm^2^/s).

**Fig. 5.**
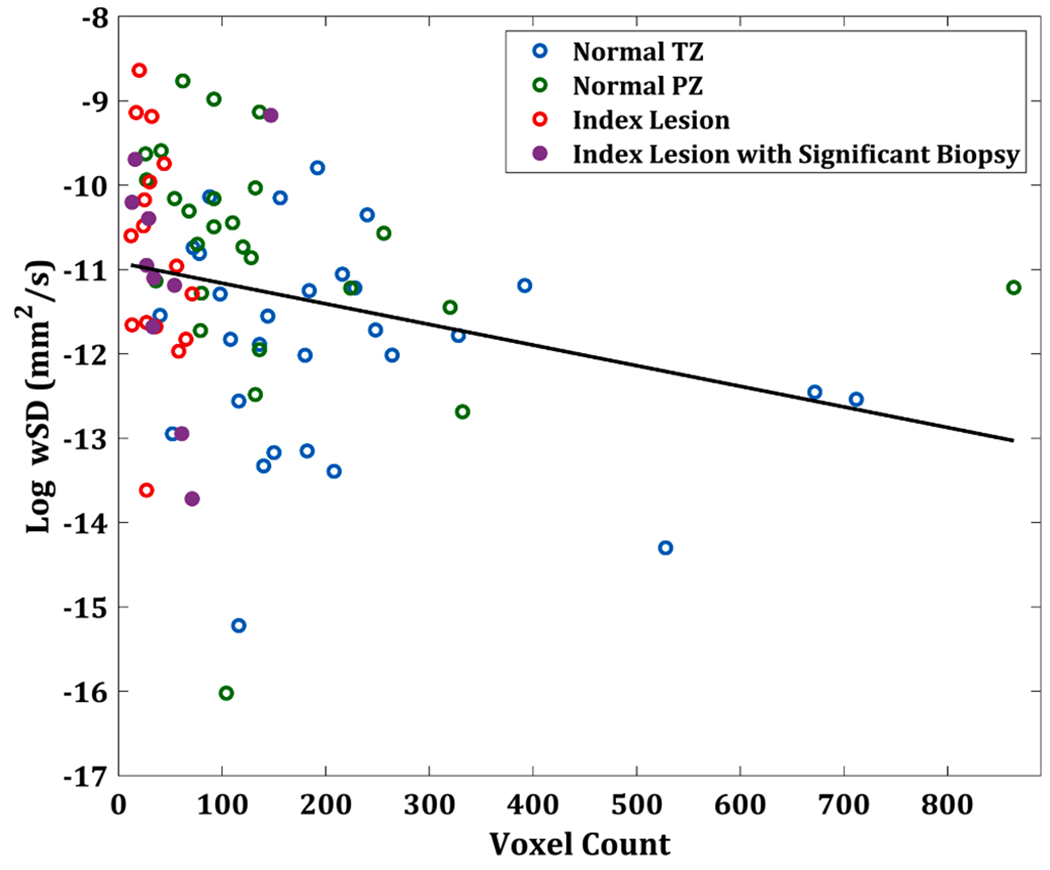
Pooled data from ROIs from 30 normal transition zone, 26 normal peripheral zone and 26 index lesions (of which 10 had significant cancer confirmed from biopsy) from 31 unique subjects. This demonstrates the relationship between number of voxels within the ROIs and the log wSD (within Standard Deviation) for ADC (Apparent Diffusion Coefficient) maps generated from *b*_0_ *b*_1500_ s/mm^2^. Generalised estimating equations (solid line) and a Wald test indicated that there was a significant relationship (*p* = 0.019) with the adjusted regression coefficient of −1.6 x10^−3^ (CI: [−3, −0.3] x10^−3^ . The region-specific measurements are colour-coded in the legend.

Pooling the data from all regions, a GLM showed that the size of the ROI was related to the wSD (*p* = 0.02). It can be seen from Fig. 5 that as the number of voxels increases, the log wSD decreases with the adjusted regression coefficient of −1.6 x10^−3^ with 95% CIs of [−3, −0.3] x10^−3^. This was close to the −1.4 x10^−3^ regression value that would be expected for random noise dependence on number of samples ($\frac{1}{\mathrm{VoxelCount}}$}. The ROI color-coding, based on zone shows that index lesions tended to have the lowest voxel counts (clustering below 100 voxels), followed by normal PZ and normal TZ, with the largest region sizes.

## 4. Discussion

ADC forms part of the mpMRI protocol and has proved valuable in prostate cancer detection and grading[21,22]. The VERDICT protocol has demonstrated utility in prostate cancer grading[16] and also allows generation of ADC surrogate metrics as the sequence consists of multiple *b*-values with variable diffusion times. Given the dependence on DWI scan parameters, in the current PI-RADS recommendations, which have been rather broad, derived ADC has only been used qualitatively. Recommendations do not include specific guidance on mixing diffusion and echo times outside of utilising TE ≤ 90 ms. Determining which *b*-value range provides repeatable ADC is important if the quantitative value is to be used for discrimination of clinically-significant cancer from benign changes and low-grade disease [3].

This study compared ADC repeatability (quantified by wSD) from different *b*-value combinations in prostate tissue using the multi *b*-value VERDICT sequence with variable diffusion times, for subjects who were scanned twice. This study provides the largest repeatability cohort (n = 41) published to date, sufficient [13] to allow analysis of repeatability dependence on acquisition parameters, region size and location. Image quality was deemed adequate for all ROIs included in the final analysis. Although VERDICT *b*-values do not include 800–1000 s/mm^2^ as recommended by PI-RADS, the ADC repeatability trends derived for lower and higher *b*-values may inform both future repeatability investigations and, ultimately, technical acquisition and analysis recommendations.

Among the five studied *b*-value combinations, the repeatability was best for ADC from *b*_0_ *b*_1500_, and was unaffected by ADC magnitude, location, and lesion Likert score. There was a relationship with ROI size; an increase in number of voxels showed a reduction in variability for normal TZ, compared to normal PZ and index lesions (with lowest sizes and highest wSD consistent with increased random noise effect on ADC). Compared to normal PZ and TZ, the observed repeatability was lower (variability higher) for index lesions and marginally better than reported in literature for protocols that used lower *b*-values and subject repositioning [10–12]. The lack of correlation of ADC repeatability with lesion Likert score for *b*_0_ *b*_1500_ also suggests that repeatability is independent of disease severity, which would allow establishing technical performance thresholds by pooling data across lesions.

It was demonstrated that ADC with *b*_*max*_ = 500 s/mm^2^ had the highest variability, indicating low repeatability. Lower repeatability observed for ADC generated from *b*_*max*_ = 500 s/mm^2^ may be attributed to perfusion effects. At lower *b*-values the diffusion signal is influenced by fast pseudo-diffusion and pulsatile perfusion effects [23]. The PI-RADS recommendations acknowledge that perfusion information may be obtained using *b*-values: 0–500 [24]. The pseudo-diffusion coefficient attributed to this relationship has previously been shown to have poor repeatability and high variability in various organs, including prostate [25–28]. Furthermore, *b*_500_ may not provide sufficient contrast-to-noise for slow diffusion in prostate tissue at moderately long diffusion times used by the VERDICT protocol.

Larger ROI sizes increased repeatability, possibly due to reduced noise effects on the mean ADC and reduced registration errors between the two scans, as a greater areas and voxel ADC samples were analysed [29]. Therefore, care should be taken when analysing smaller lesions, especially in the PZ where most cancerous lesions are sited.

To reduce kurtosis effects, seen at higher *b*-values, PI-RADS recommends using *b*_*max*_ = 1000 for ADC calculations [2]. VERDICT does not include *b*_1000_, however, the present study is still in line with QIBA guidelines, which recommend *b*_*max*_ = 500–1500 [5]. To reduce variability of multi *b*-value acquisitions and comply with mono-exponential ADC model assumptions, it is essential to use consistent TEs, which was not possible for retrospective analysis of VERDICT acquisitions in this study. To alleviate inconsistent TE, the studied *b*-value combinations were limited to those within 15 ms difference. Adhering to PI-RADS recommendations (TE ≤ 90 ms), shorter diffusion times for high *b*-values would also help reduce SNR bias in derived mean ADC values.

An intrinsic limitation of this study is that VERDICT uses different TRs and TEs for different *b*-values which may not be optimised for prostate ADC measurement. Mean ADC values were highly variable across the different *b*-value combinations. Only *b*_2000_ has TR ≥ 3,000 ms, which is the current PI-RADS and QIBA recommendation [2,5]. A longer TR is needed to alleviate T_1_ effects for ADC; however, VERDICT is optimised for prostate cancer detection and grading based on the model that quantifies extra- and intra-cellular diffusion fractions, rather than ADC [15]. As VERDICT is clinically promising [16] it was beneficial to investigated whether this sequence could also generate repeatable ADC maps without modifying acquisition parameters, and retrospectively selecting *b*-value combinations with close TEs.

The lower variability observed for the higher *b*-value combinations may be related to increased *b*-value averaging, which is needed due to the decrease in signal with increase in *b*-value (*b*_90_ = 4 averages, *b*_0_, *b*_500_, *b*_1500_ and *b*_2000_ = 6 averages). This may not be practical for clinical protocols due to the increased scan time. Additionally, high repeatability does not guarantee clinical diagnostic value. However, utilising > *b*_1000_ for ADC has shown promise in discrimination between high and low grade prostate tumours [9]. Our results indicated marginal improved correlation of ADC with Likert score for *b_0_ b_500_ b_2000_* than *b_0_ b_1500_*, which had better repeatability. The clinical choices of ADC acquisition will need to balance improved stability and improved discriminatory power.

Another limitation, due to the retrospective nature of the analysis, is that the test–retest scans were performed on a single system during a single exam with majority of patients not undergoing repositioning. This only provided short-term ADC repeatability values. These errors could be optimistic for long-term or multi-site prostate ADC studies. However, other research (using lower maximum *b*_*max*_ = 800 s/mm^2^) has shown that test-retest repeatability with repositioning and multi-day reproducibility are largely equivalent [30]. Furthermore, acquired data was sufficient for the purpose of the present study to evaluate relative repeatability trends across *b*-value protocols, prostate zones, region sizes, and ADC values. Additionally, the subset of patients who were repositioned showed similar repeatability results and thus added support for assessed repeatability values. Repeatability of index lesions was similar to that observed for significant cancers confirmed by biopsy.

In summary, ADC maps formed from *b*_0_ *b*_1500_ combination from the VERDICT acquisition had the least variability. This was found in normal TZ, normal PZ, and prostate cancer lesions. Repeatability increased with ROI size and for normal TZ, versus normal PZ, versus index lesion consistent with reduced noise for mean ADC. The index lesion mean ADC was moderately negatively correlated with Likert score. Importantly, lesion wSD did not increase with ADC magnitude, hence wSD was a more appropriate repeatability metric than wCV (normalised by mean ADC) for prostate ADC. Future studies should address the limitation of consistent acquisition parameters to optimize ADC acquisitions and evaluate effect of scan resolution for small lesions. Having low variability makes it easier to ascertain whether there have been clinically significant ADC changes in longitudinal therapy response studies and active surveillance prostate imaging programs.

## Supplementary Material

SuplmentaryFigs

## Figures and Tables

**Table 1:** Patient demographics.

	Non-Repositioned Cohort (n = 31)	Repositioned Cohort (n = 10)
**Median Age (years)**	67	68
**Age Range(years)**	50 – 79	50 – 81
**Median Serum PSA (ng/ml)**	8.96	6.25
**Serum PSA Range (ng/ml)**	2.25 – 30.93	2.50 – 19.83

Note. – PSA = Prostate-Specific Antigen.

**Table 2:** The VERDICT MRI acquisition.

G mT/m	Δ (ms)	δ (ms)	b values/mm^2^	TE (ms)	TR (ms)	NEX	Acquisition Time (seconds)
61.2	23.8	3.9	90	50	2482	4	42
44.4	31.3	11.4	500	65	2482	6	107
32.1	43.8	23.9	1500	90	2398	6	146
80.5	32.3	12.4	2000	67	3897	6	237
60.2	38.8	18.9	3000	80	3349	6	204

Note. – G = gradient strength, Δ = timing between gradients, δ = gradient pulse duration, TE = echo time, TR = repetition time, NEX = number of excitations.

**Table 3:** The 4*b*-value combinations used to form the ADC maps.

ID of Range	*b*-value Combination (s/mm^2^)
1	0 500
2	0 1500
3	0 2000
4	90 500
5	0 500 2000

Note. – ADC = Apparent Diffusion Coefficient.

**Table 4:** Mean wSD and Mean ADC and Standard Deviations for each *b*-value combination in the different locations for the non-repositioned cohort (n = 31) and the repositioned cohort (n = 10).

*b*-value Combination s/mm^2^	Mean wSD [95% CIs] (x10^−4^ mm^2^/s)	Mean ADC (SD) (x10^−4^ mm^2^/s)	Mean wSD [95% CIs] (x10^−4^ mm^2^/s)	Mean ADC (SD) (x10^−4^ mm^2^/s)	Mean wSD [95% CIs] (x10^−4^ mm^2^/s)	Mean ADC (SD) (x10^−4^ mm^2^/s)
**Non-Repositioned**	**Normal TZ (n = 30)**		**Normal PZ (n = 26)**		**Index Lesion (n = 26)**	
0 500	3.08 [2.46, 4.12]	17.14 (3.65)	7.94 [6.25, 10.88]	25.14 (5.77)	8.72 [6.87, 11.95]	18.52 (7.00)
0 1500	0.18 [0.14, 0.24]	3.8 (0.50)	0.53 [0.42, 0.73]	4.46 (0.69)	0.56 [0.44, 0.76]	3.66 (0.68)
0 2000	0.23 [0.18, 0.30]	6.74 (0.80)	0.57 [0.45, 0.79]	7.63 (1.31)	0.83 [0.66, 1.14]	6.12 (1.28)
90 500	1.9 [1.52, 2.54]	20.75 (1.88)	7.15 [5.63, 9.79]	25.22 (3.97)	6.54 [5.15, 8.97]	22.6 (4.59)
0 500 2000	0.34 [0.27, 0.45]	8.32 (1.22)	1.02 [0.80, 1.40]	9.85 (1.72)	1.17 [0.93, 1.61]	7.71 (1.75)
**Repositioned**	**Normal TZ (n = 7)**		**Normal PZ (n = 4)**		**Index Lesion (n = 9)**	
0 500	4.5 [2.98, 9.16]	21.14 (4.18)	7.63 [4.57, 21.94]	26.84 (7.22)	10.24 [7.04, 18.69]	23.72 (9.33)
0 1500	0.43 [0.28, 0.87]	4.25 (0.31)	0.4 [0.24, 1.15]	4.54 (0.85)	0.58 [0.40, 1.06]	3.48 (0.68)
0 2000	0.29 [0.19, 0.59]	7.3 (0.73)	0.28 [0.17, 0.82]	7.76 (1.54)	1.79 [1.23, 3.27]	5.78 (1.03)
90 500	3.27 [2.16, 6.65]	22.95 (2.46)	6.44 [3.86, 18.51]	30.13 (5.58)	9.53 [6.56, 17.40]	25.85 (7.29)
0 500 2000	0.83 [0.55, 1.69]	9.32 (0.67)	0.42 [0.25, 1.20]	9.78 (1.84)	1.08 [0.75, 1.98]	7.42 (1.40)

Note. – wSD = within-subject standard deviation, ADC = Apparent Diffusion Coefficient, SD = Standard Deviation, 95% CIs = Confidence Intervals, TZ = Transition Zone, PZ = Peripheral Zone.

## Abbreviations:

ADC: Apparent Diffusion Coefficient
VERDICT: Vascular Extracellular and Restricted Diffusion for Cytometry in Tumors
PI-RADS: Prostate Imaging Reporting and Data System
ROI: Region of Interest
mpMRI: Multiparametric MRI
wSD: Within-subject Standard Deviation
TZ: Transition Zone
PZ: Peripheral Zone
DWI: Diffusion Weighted Imaging
