# Supplementary material for: Test-retest repeatability of ADC in prostate using the multi b-Value VERDICT acquisition
Source: Eur J Radiol. Author manuscript; Available in PMC 2023 Jul 11. (PMC10334409; doi:10.1016/j.ejrad.2023.110782)
Supplement: SuplmentaryFigs [file NIHMS1911486-supplement-SuplmentaryFigs.docx]

**Supplementary Figures**

**
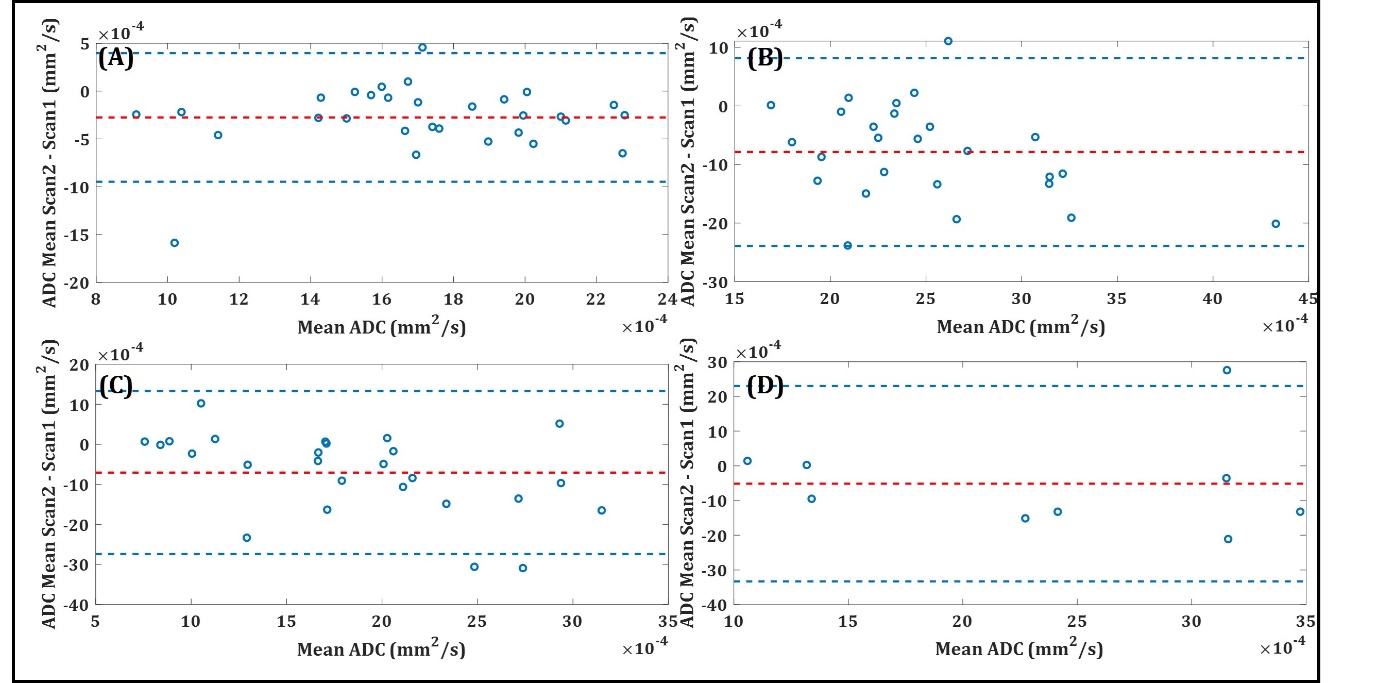
**

**Figure S1.** Bland-Altman analyses showing the level of agreement of mean ADC (Apparent Diffusion Coefficient) between scan 1 and scan 2. ADC generated from *b*_0_ *b_500_* s/mm^2^. Blue dotted lines indicate the confidence intervals (1.96*standard deviation). The Confidence Intervals (CI) for each of the analysed regions are provided **(A)** Normal Transition Zone for non-repositioned cohort, (CI: [2.46, 4.12] x10^-4^ mm^2^/s) **(B)** Normal Peripheral Zone for non-repositioned cohort, (CI: [6.25, 10.88] x10^-4^ mm^2^/s) **(C)** Index Lesion for non-repositioned cohort, (CI: [6.87, 11.95] x10^-4^ mm^2^/s) **(D)** Index Lesions for repositioned cohort, (CI: [7.04,18.69] x10^-4^ mm^2^/s).

**
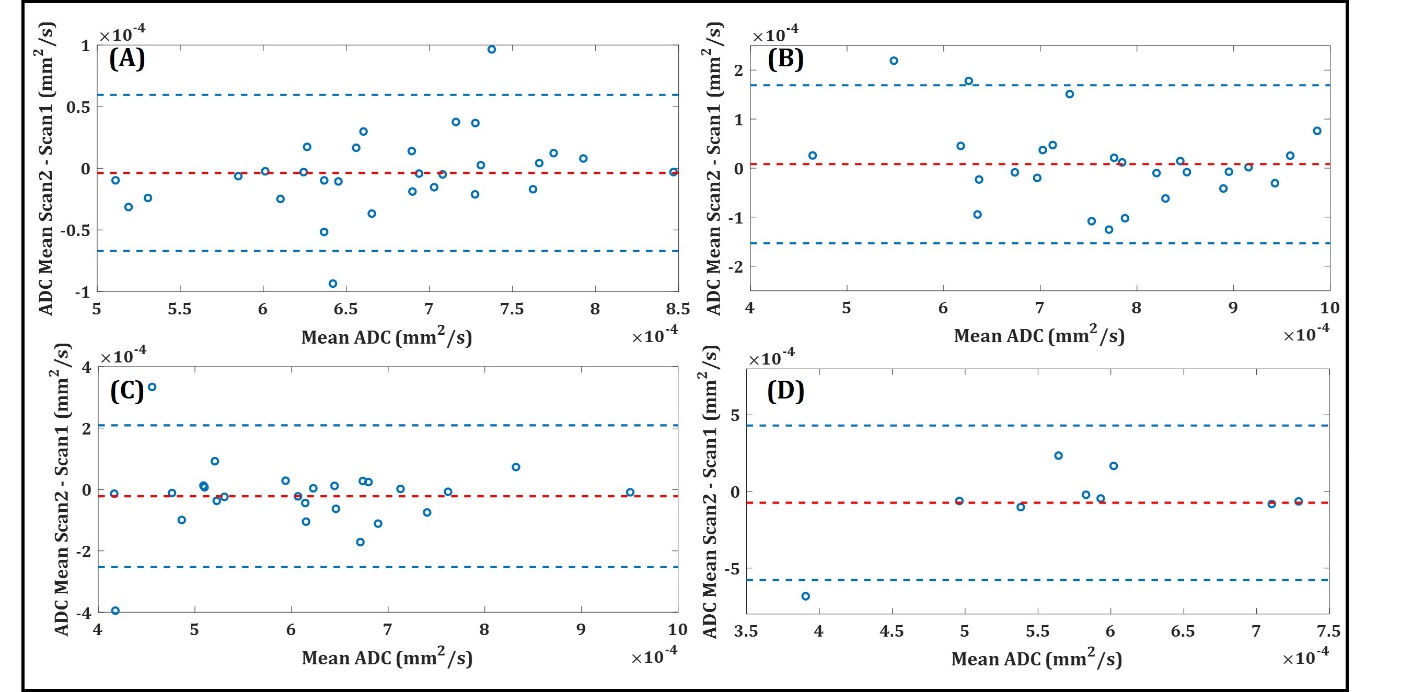
**

**Figure S2.** Bland-Altman analyses showing the level of agreement of mean ADC (Apparent Diffusion Coefficient) between scan 1 and scan 2. ADC generated from *b*_0_ *b_2000_* s/mm^2^. Blue dotted lines indicate the confidence intervals (1.96*standard deviation). The Confidence Intervals (CI) for each of the analysed regions are provided: **(A)** Normal Transition Zone for non-repositioned cohort, (CI: [0.18, 0.30] x10^-4^ mm^2^/s) **(B)** Normal Peripheral Zone for non-repositioned cohort, (CI: [0.45, 0.73] x10^-4^ mm^2^/s) **(C)** Index Lesion for non-repositioned cohort, (CI: [0.66, 1.14] x10^-4^ mm^2^/s) **(D)** Index Lesions for repositioned cohort, (CI: [1.23, 3.27] x10^-4^ mm^2^/s).


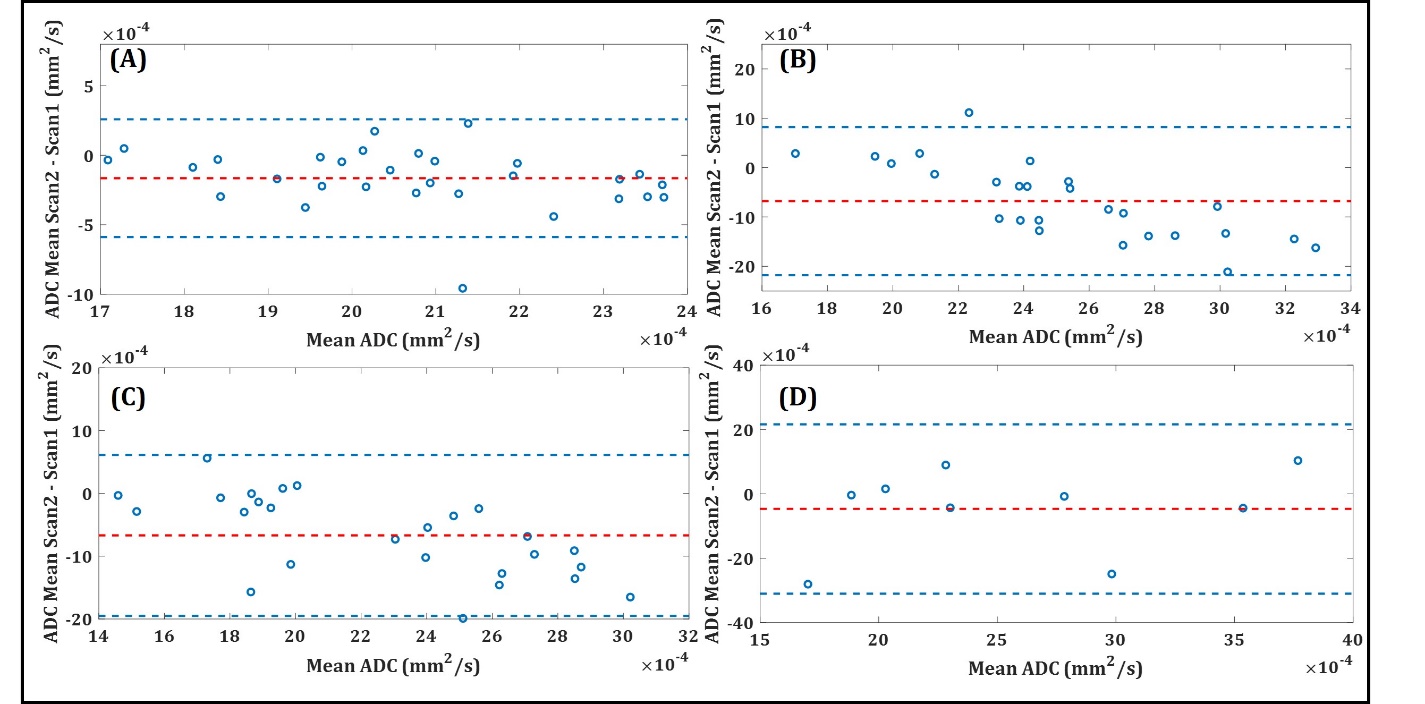


**Figure S3.** Bland-Altman analyses showing the level of agreement of mean ADC (Apparent Diffusion Coefficient) between scan 1 and scan 2. ADC generated from *b*_90_ *b_500_* s/mm^2^. Blue dotted lines indicate the confidence intervals (1.96*standard deviation). The Confidence Intervals (CI) for each of the analysed regions are provided: **(A)** Normal Transition Zone for non-repositioned cohort, (CI: [1.52, 2.54] x10^-4^ mm^2^/s) **(B)** Normal Peripheral Zone for non-repositioned cohort, (CI: [5.63, 9.79] x10^-4^ mm^2^/s) **(C)** Index Lesion for non-repositioned cohort, (CI: [5.15, 8.97] x10^-4^ mm^2^/s) **(D)** Index Lesions for repositioned cohort, (CI: [6.56, 17.40] x10^-4^ mm^2^/s).


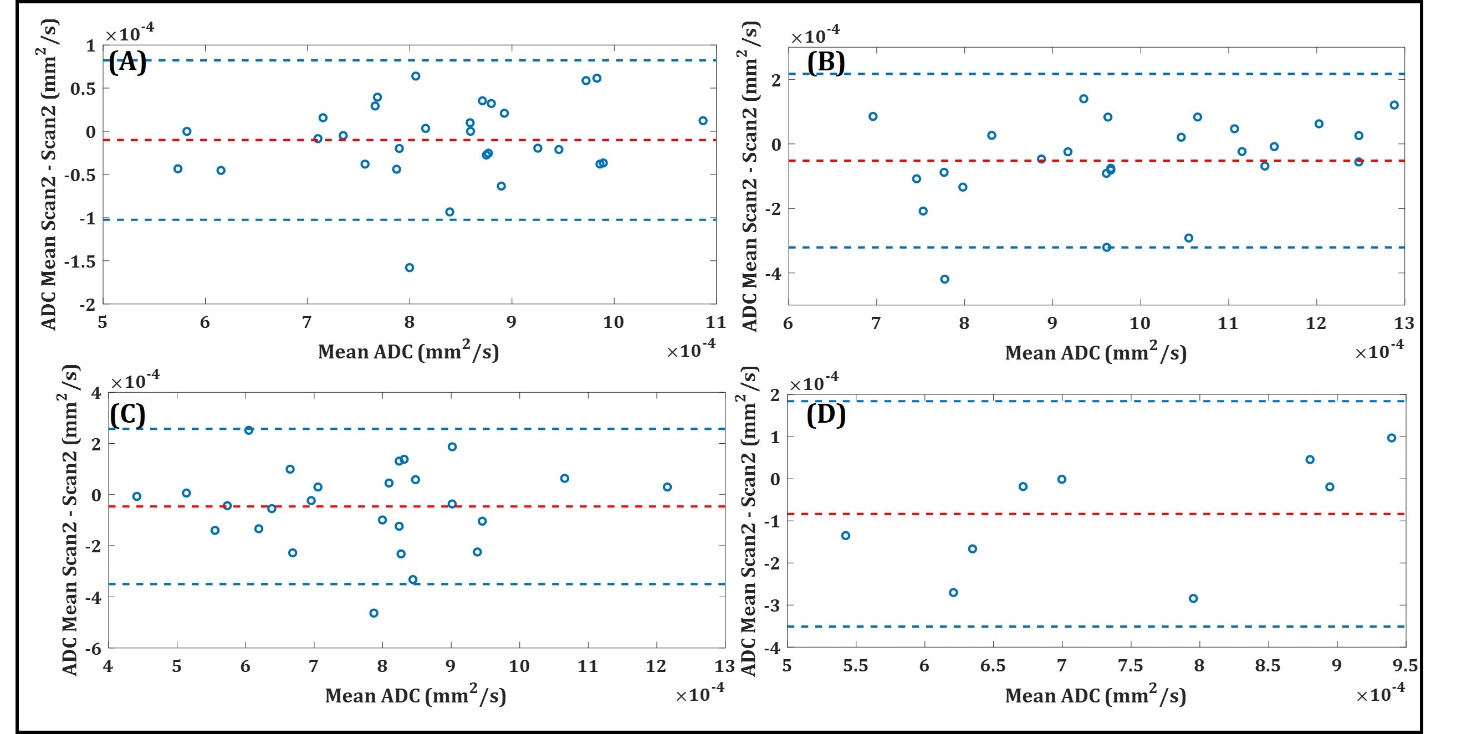


**Figure S4.** Bland-Altman analyses showing the level of agreement of mean ADC (Apparent Diffusion Coefficient) between scan 1 and scan 2. ADC generated from *b*_0_ *b_500_* *b_2000_* s/mm^2^. Blue dotted lines indicate the confidence intervals (1.96*standard deviation). The Confidence Intervals (CI) for each of the analysed regions are provided: **(A)** Normal Transition Zone for non-repositioned cohort, (CI: [0.27, 0.45] x10^-4^ mm^2^/s) **(B)** Normal Peripheral Zone for non-repositioned cohort, (CI: [0.80, 1.40] x10^-4^ mm^2^/s) **(C)** Index Lesion for non-repositioned cohort, (CI: [0.93, 1.61] x10^-4^ mm^2^/s) **(D)** Index Lesions for repositioned cohort, (CI: [0.75, 1.98] x10^-4^ mm^2^/s).
